# Persistent Cognitive Dysfunction in Postural Orthostatic Tachycardia Syndrome: A Scoping Review of Neural Mechanisms, Interventions, and Critical Research Gaps

**DOI:** 10.7759/cureus.112862

**Published:** 2026-07-17

**Authors:** Ananya Rao Prassanna

**Affiliations:** 1 Orthopaedics, Independent Research, Apex, USA

**Keywords:** autonomic dysregulation, cerebral hypoperfusion, cognitive dysfunction, neuroinflammation and autoimmunity, postural orthostatic tachycardia syndrome (pots)

## Abstract

Postural orthostatic tachycardia syndrome (POTS) is widely treated as a cardiovascular-autonomic syndrome, but cognitive dysfunction is a disabling and poorly treated clinical phenomenon. This scoping review discusses why brain fog, impaired attention, slowed processing speed, memory difficulties, word-finding issues, and executive dysfunction may persist despite conventional pharmacological and non-pharmacological POTS treatments. The study uses a structured scoping review design to synthesize 18 peer-reviewed sources that meet the inclusion criteria, with publication dates between 2013 and 2025, across the domains of mechanistic, clinical, treatment, rehabilitation, and interventional studies. The evidence can be grouped into three principal mechanistic pathways: cerebral hypoperfusion and hemodynamic dysfunction; autonomic dysregulation and sympathetic overactivation; and neuroinflammation, autoimmune responses, mast cell activation, and microclot formation. The review found that abnormal cerebral blood flow (CBF) was reported in 61% of 56 POTS patients with cognitive dysfunction, and that sustained cognitive stress induced a greater reduction in CBF velocity in POTS patients than in controls, at approximately 7.8% and 1.8%, respectively. Current therapies, including beta-blockers, ivabradine, midodrine, exercise, salt and fluid loading, compression garments, cognitive behavioral therapy (CBT), and sleep management, rarely use cognition as a primary endpoint. Findings suggest that persistent cognitive dysfunction in POTS may reflect incomplete alignment between neurological mechanisms and cardiovascular-centered care, requiring validated cognitive outcomes and multimodal trials combining biological, autonomic, and rehabilitative interventions.

## Introduction and background

Postural orthostatic tachycardia syndrome (POTS) is a chronic dysregulation of the autonomic nervous system (ANS) that causes inappropriate tachycardia and symptoms upon standing, such as chest pain, shortness of breath, fatigue, lightheadedness or fainting, nausea, and cognitive dysfunction. Typically, the syndrome is diagnosed based on an abnormal increase in heart rate in response to an orthostatic challenge, but it is also associated with considerable functional impairment. POTS predominantly affects premenopausal women, especially those between 15 and 45 years old, and is therefore particularly important in terms of education, career, parenting, and long-term productivity [1]. In this group, cognitive dysfunction is consistently reported as one of the most bothersome but poorly treated aspects of POTS.

The impacts of cognitive dysfunction in POTS are often described as foggy-headedness or brain fog but represent multiple deficits. These include poor sustained attention, working memory, processing speed, word-finding, reaction time, and executive function. These problems can affect reading, learning, driving, decision-making at work, and completing daily tasks. Current management of POTS often focuses on orthostatic symptom control and cardiovascular-autonomic targets. This includes increased fluid and salt intake, progressive exercise training, compression stockings, beta-blockers, ivabradine, and midodrine. These treatments can help with orthostatic symptoms and heart rate control but are not specifically aimed at improving cognitive performance.

The key issue in the current study is that while improvements in heart rate, blood pressure, and orthostatic symptoms are observed, cognitive symptoms may persist. This suggests that the cognitive symptoms of POTS are not merely a “downstream” side effect of tachycardia. Rather, the existing evidence suggests that brain fog may be caused by one or more of several simultaneous neurological, cerebrovascular, autonomic, inflammatory, and autoimmune factors. Brain hypoperfusion, impaired neurovascular responses, sympathetic hyperactivation, baroreflex dysregulation, autoantibody activity, mast cell-mediated inflammation, and fibrinaloid microclot formation may all contribute to brain fog and executive dysfunction.

Despite this range of possible mechanisms, POTS has previously been defined and approached primarily as a dysfunction of cardiovascular control. As a result, trials of POTS treatment tend to focus on primary outcomes of heart rate reduction and orthostatic tolerance, as well as other symptoms and physical quality of life [2, 3]. Cognitive outcomes are either absent, secondary, or inconsistently reported. This poses an operational problem: disease processes likely to underpin cognitive deficits include neurological and inflammatory mechanisms, yet interventions are assessed primarily for their cardiovascular effects. One limitation of this study is the lack of a validated POTS-specific cognitive endpoint questionnaire. Without such a tool, it is difficult to determine whether an intervention improves cognition, worsens cognitive deficits, or offers cardiovascular benefits without significant neurocognitive improvement. In practice, this results in suboptimal educational performance, lost productivity, reduced work performance, loss of independence, and a decrease in quality of life.

The central research question guiding this review is: Why do cognitive symptoms persist despite standard pharmacological and non-pharmacological treatments that primarily target cardiovascular or autonomic outcomes, and could cognition-targeted interventions address this gap? This review will analyze patterns in reported cognitive outcomes, assess the frequency and quality of cognitive measures used in intervention studies, and determine whether existing research has identified a viable cognition-targeted therapeutic approach.

The present article will concentrate on persistent cognitive impairment in POTS itself, rather than all manifestations of POTS. It identifies three possible mechanisms: brain hypoperfusion and hemodynamic dysfunction; hyperadrenergic signaling and autonomic dysfunction; and neuroinflammation, autoimmunity, mast cell activation, and fibrinaloid microclots. It also outlines the cognitive limitations of pharmacological and non-pharmacological interventions, along with treatments that have demonstrated efficacy and may also have cognitive benefits. The research does not support the notion that a single mechanism is responsible for the development of cognitive symptoms, since POTS is a diverse syndrome with possible hypovolemic, neuropathic, hyperadrenergic, autoimmune, and post-viral subtypes.

This work contains several sections. The Literature Review analyzes mechanisms, treatments, psychosocial problems, and research gaps. The Methods and Techniques section describes the review process, data sources, selected studies, analysis approach, and quality evaluation. The Results and Discussion section explains the main findings and how they may influence future work. The review provides practical suggestions for future research on measuring outcomes and designing interventions. The study concludes by recommending a brain- and cognition-oriented research approach to POTS research endeavors. Cognitive dysfunction is not a peripheral feature of POTS but its most functionally disabling feature for many patients. Treating it accordingly is not an expansion of the field's scope but a correction of its most consequential omission.

This article is presented as a scoping review because its primary objective is to map current evidence regarding mechanisms, interventions, and knowledge gaps related to persistent cognitive dysfunction in POTS rather than to perform quantitative evidence synthesis. The review integrates mechanistic studies, observational investigations, clinical reports, rehabilitation literature, and systematic reviews to provide a comprehensive overview of the field while identifying priorities for future research.

## Review

Cerebral hypoperfusion and hemodynamic dysfunction

Cerebral hypoperfusion is a measurable phenomenon and may explain how POTS can induce persistent cognitive impairment by reducing blood flow that supplies oxygen and nutrients to cortical neuronal networks. The hemodynamic model showed that a 30% drop in blood volume could reduce cerebral blood flow (CBF) by about 100 mL/min. Comparatively, a 50-100% increase in arterial stiffness would tend to decrease cardiac output and cerebral perfusion, since tachycardia can both contribute to hemodynamic strain and serve as an offsetting response to maintain blood flow [4]. From a neuroimaging perspective, another study applied brain single-photon emission computed tomography (SPECT) imaging to 56 POTS patients with cognitive dysfunction and revealed abnormal CBF in 61% even while supine, a result that dissociates cerebral hypoperfusion from orthostatic posture alone. Attention and executive function are associated with the lateral regions of the prefrontal cortex, while sensorimotor abnormalities may contribute to impaired processing speed and sensorimotor integration. Prolonged cognitive stress also seems prominent; POTS patients showed a 7.8% reduction in CBF velocity during a cognitive task compared to 1.8% in controls, while SPECT findings also suggest that POTS patients may be vulnerable to abnormal cerebral perfusion even outside orthostatic stress [5]. These findings indicate that more effective clinical workflows that include orthostatic vital signs, cognitive tests, brain perfusion factors, fatigue self-reporting, and pulse changes would be more useful.

The relationships among mean arterial pressure, intracranial pressure, cerebral perfusion pressure, and CBF are relevant to this mechanism. This mechanism is consistent with cerebral hypoperfusion in POTS, in which reduced effective perfusion may inhibit the supply of oxygen and nutrients to cortical networks. In cases of continued cognitive dysfunction, disruption of CBF may affect attention, executive function, processing speed, and cognitive-motor performance in ways that are not directly posture-dependent.

Autonomic dysregulation, baroreflex failure, and sympathetic overactivation

Autonomic dysfunction plays a role in cognitive dysfunction through sympathetic hyperactivation, catecholaminergic shifts, vascular dysfunction, and cardiovagal buffering. In a case-control study that recruited 87 patients with POTS and 39 controls, Miller AJ et al. found reduced executive function in POTS patients (t-scores: 48 +/- 11 in POTS vs 55 +/- 10 in controls, p = 0.009) and impaired attention and reaction speed when standing [6]. This evidence suggests an underlying dysfunction that worsens with upright posture.

Mueller BR et al. also associated decreased cardiovagal baroreflex sensitivity with the diagnosis of POTS, with an adjusted odds ratio of 5.78 (p = 0.047; CI = 1.0-32.5), although the wide CI indicates limited precision [7]. Overstimulated prefrontal circuits may respond to noradrenergic overarousal due to poor cardioregulation, leading to executive dysfunction. The literature also reports overlapping cognitive and psychological challenges in POTS and supports a scenario in which anxiety, depression, vigilance, and autonomic arousal may interact with symptoms.

Neuroinflammation, autoimmunity, mast cell activation, and microclots

Another literature stream describes prolonged cognitive dysfunction as, in part, inflammatory and autoimmune, which is notable because cardiovascular drugs may not directly address immune-related brain mechanisms. Researchers speculate that fibrinaloid microclots seen in POTS, particularly long-COVID-associated POTS, may lead to microcapillary obstruction and local tissue hypoxia. Kell DB et al. (2024) proposed that amyloid proteins may affect nerve membranes and contribute to neurotoxicity and ANS dysfunction through membrane disruption, supporting the possibility of neurological dysfunction through amyloid-related membrane and vascular effects. Kesterson K et al. (2023) also reported improvement in symptoms with subcutaneous immunoglobulin or plasmapheresis in patients with presumed autoimmune POTS and suggested that immunotherapy may benefit a subset of patients rather than all phenotypes [8]. There were also reported improvements in cognition after plasma exchange treatment in patients with autoimmune neurological dysfunction and long-COVID-related POTS, providing direct but limited proof of concept for cognition-sensitive immunotherapy. Mast cell activation has been less well quantified but is also significant in practice, as the release of inflammatory cytokines may exacerbate vascular tone abnormalities, tachycardia, headaches, and brain fog symptoms in some patients.

Current treatment approaches and cognitive limitations

Pharmacological evidence remains dominated by cardiovascular measures. Pierson BC et al. (2025) found, in a systematic review of 32 studies, that among the oral drugs studied, beta-blockers, ivabradine, and midodrine were the most common, with benefits generally measured by reductions in heart rate, orthostasis, or global improvement, but not cognitive function [9]. The clinical importance of this neuroinflammatory and autoimmune axis is that it represents a mechanistic pathway identified in this review that is not directly targeted by frontline pharmacological interventions. Beta-blockers, ivabradine, and midodrine are not primarily anti-inflammatory, anticoagulant, or immunomodulatory therapies, which may help explain why cognitive symptoms persist in some patients whose orthostatic tachycardia has been pharmacologically regulated. Non-pharmacological treatment has several advantages. Researchers emphasize interventions to treat headache and other chronic pain through exercise, sleep, behavioral treatment, and other supportive measures, but these interventions are aimed at treating pain, catastrophizing, sleep disturbance, and somatic amplification processes rather than memory, attention, and executive functions [10].

In another study, 149 individuals under investigation for POTS were tracked over a period of six months. The study found persistently high levels of symptomatology, with 48% of the baseline variance and 4% of the six-month variance being explained by psychosocial variables and hemodynamic factors, respectively [11]. A new systematic review of symptom burden also notes that results from studies employing longitudinal or interventional designs are inconsistent, making them difficult to compare. Thus, existing therapy may help treat orthostatic symptoms but not cognitive abilities.

Table 1 provides an overview of current patterns of POTS treatment and their cognitive constraints, with pharmacological interventions, including beta-blockers, ivabradine, and midodrine, primarily discussed with respect to their effects on heart rate, control of orthostatic symptoms, and overall improvement. It also points out that non-pharmacological interventions, such as exercise, sleep management, behavioral therapy, and supportive care, often address pain, sleep disturbance, behavioral factors, and supportive care needs instead of memory, attention, or executive functioning. The table highlights the persistent absence of overlap between orthostatic control and measurable cognitive restoration in the practice of POTS care, as evidenced by longitudinal and symptom burden data in real clinical practice.

**Table 1:** Current POTS treatment approaches, measured endpoints, and cognitive limitations. POTS: Postural orthostatic tachycardia syndrome.

Treatment category	Specific approaches discussed	Main measured outcomes	Cognitive limitation	Source(s)
Pharmacological treatment	Beta-blockers, ivabradine, and midodrine	Heart rate reduction, orthostatic symptom control, and general symptom improvement across treatment studies	Cognitive recovery is rarely measured; beta-blockers may also worsen fatigue or cognitive slowing in some patients	[9]
Non-pharmacological treatment	Exercise, sleep management, behavioral therapy, pain management, and supportive strategies	Headache control, chronic pain management, sleep improvement, reduced catastrophizing, and reduced somatic amplification	These interventions mainly target pain, sleep, behavioral symptoms, and general symptom burden, rather than memory, attention, or executive function	[10]
Longitudinal symptom monitoring	Six-month follow-up of 149 people under investigation for POTS	Symptom burden remained high; psychosocial factors explained 48% of baseline variance, while hemodynamic measures explained 4% of six-month variance	Persistent symptoms suggest that standard care may not produce measurable cognitive restoration over time	[11]
Outcome measurement in POTS studies	Treatment and longitudinal symptom-monitoring studies	Outcomes are commonly focused on heart rate, orthostatic symptoms, pain, sleep, psychosocial variables, or general symptom burden	The lack of standardized cognitive endpoints makes it difficult to determine whether treatments improve cognition	[9-11]

Research gaps, digital measurement, and rehabilitation needs

The major research gaps involve not only a deficit in treatment but also a deficit in cognition-focused measurement. Hollingsworth C et al. (2022) showed that patients report impaired memory, sustained attention, word-finding, and executive processing, and that these difficulties are occupationally limiting. Patients independently apply digital memory devices, task simplification, pacing, and environmental simplification to compensate [12]. These strategies resonate with occupational therapy treatment but remain untested in randomized controlled trials for POTS. Digital infrastructure can also help fill this gap, provided it is applied thoughtfully. Research offers examples of applying AI-powered healthcare analytics to security, personalization, and compliance, which could be relevant to future POTS registries that collect data on cognition, autonomic functioning, biomarkers, and responses to treatments at scale [13].

The analysis of healthcare-related AI-driven call-center automation showcases how it improves efficiency, which provides a real-world example of symptom-based triage, scheduling follow-ups, and monitoring disease remotely in chronic illnesses [14]. Future research on POTS should address the following subtypes: hypovolemic, neuropathic, hyperadrenergic, autoimmune, and post-viral; support the use of cognitive outcomes; and explore multimodal therapy combining immune therapy, autonomic regulation, and occupational rehabilitation. This would enable the literature to be applied to specialized clinics and rehabilitation programs, as well as to longitudinal digital care solutions. These designs would also facilitate payer care management, patient care monitoring, interdisciplinary care support, and personalization of treatment.

Methods and techniques

Data Collection Methods

This section provides a structured scoping review of persistent cognitive dysfunction in POTS, focusing on mechanisms, intervention strategies, and research gaps. Data sources included biomedical, neurological, autonomic, cardiovascular, rehabilitation, and clinical-trial literature. The databases searched included PubMed/MEDLINE, Scopus, Web of Science, Google Scholar, the Cochrane Library, and ClinicalTrials.gov. Keywords used included POTS-related terms and cognitive, mechanistic, and intervention-related terms such as “POTS cognitive dysfunction,” “POTS brain fog,” “postural tachycardia syndrome cerebral blood flow,” “POTS cerebral hypoperfusion,” “POTS autonomic dysregulation cognitive cycle,” “POTS neuroinflammation autoimmunity,” “POTS vagus nerve stimulation,” and “POTS IVIG plasma exchange.” The search strategy aimed to identify objective mechanistic evidence, such as abnormal SPECT brain scans in POTS patients with cognitive dysfunction, and evidence related to treatment in clinical settings [15].

Search Strategy

A structured literature search was conducted in PubMed/MEDLINE, Scopus, Web of Science, Google Scholar, the Cochrane Library, and ClinicalTrials.gov. Search terms combined Medical Subject Headings (MeSH) and free-text keywords including “postural orthostatic tachycardia syndrome,” “POTS,” “brain fog,” “cognitive dysfunction,” “cerebral blood flow,” “SPECT,” “autonomic dysfunction,” “baroreflex,” “microclots,” “mast cell activation,” “autoimmunity,” “transcutaneous vagus nerve stimulation,” “plasma exchange,” and “intravenous immunoglobulin.” Boolean operators (AND/OR) were used to combine search concepts. Searches were limited to English-language peer-reviewed publications published between January 2013 and March 2025. Reference lists of eligible articles were also manually screened for additional studies.

**Figure 1:**
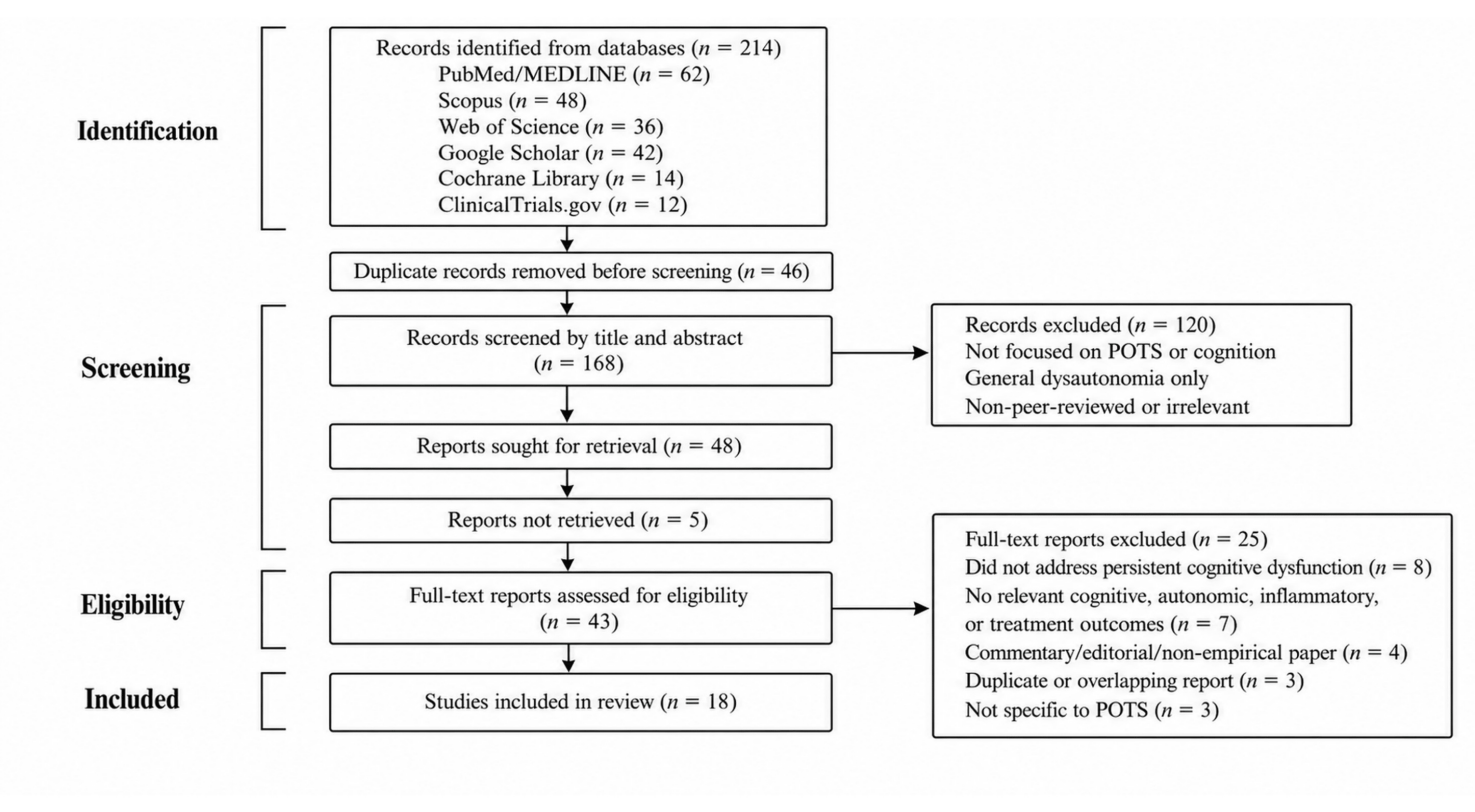
PRISMA flow diagram of study selection. Created by the author using Microsoft PowerPoint, based on the study selection process and PRISMA 2020 guidelines. PRISMA: Preferred Reporting Items for Systematic Reviews and Meta-Analyses.

The selection of studies is outlined in Figure 1. A total of 214 records were identified from PubMed/MEDLINE, Scopus, Web of Science, Google Scholar, the Cochrane Library, and ClinicalTrials.gov. After the removal of 46 duplicate records, 168 records were screened by title and abstract. From these, 120 records were excluded because they were not focused on POTS or cognition, only described general dysautonomia, or were non-peer-reviewed or irrelevant. Forty-eight reports were sought for retrieval, of which five were not retrieved. Of the remaining 43 full-text reports, 25 were excluded because they did not address persistent cognitive dysfunction, did not include relevant cognitive, autonomic, inflammatory, or treatment outcomes, were commentary/editorial/non-empirical papers, were duplicate or overlapping reports, or were not specific to POTS. A total of 18 peer-reviewed articles from 2013 to 2025 were included in the final sample for analysis.

Because the objective of this review was evidence mapping rather than quantitative synthesis, purposive criterion-based sampling was employed. Eligible studies included systematic reviews, observational studies, mechanistic investigations, interventional studies, rehabilitation research, and clinically informative case reports that examined persistent cognitive dysfunction, cerebral perfusion, autonomic dysfunction, immune mechanisms, or cognitive rehabilitation in patients with POTS. Studies without cognitive outcomes, studies focused on general dysautonomia without POTS-specific relevance, conference abstracts, editorials, duplicate publications, and articles lacking sufficient methodological information were excluded [16].

Two reviewers independently screened titles and abstracts, followed by full-text assessment of potentially eligible studies. Any disagreements regarding eligibility were resolved through discussion and consensus.

Data Analysis

The data were analyzed using a thematic approach, focusing on three categories: low CBF and hemodynamic abnormalities; autonomic dysfunction and sympathetic hyperactivity; and neuroinflammation, autoimmune responses, mast cell activation, and fibrinaloid microclots. Sources were coded according to study design, size, physiological measures, cognitive measures, intervention endpoint, and outcome. Comparative studies then explored how each intervention targeting particular processes fit the proposed mechanism. For example, noninvasive vagus nerve stimulation was classified as an autonomic modulation intervention because it increases parasympathetic tone and may correct baroreflex and sympathetic imbalances [17]. Studies on immunotherapy were coded separately because they tested immune-mediated mechanisms rather than rate-control mechanisms.

Quantitative meta-analysis was not undertaken because of substantial heterogeneity in study design, participant populations, outcome measures, intervention characteristics, and reported cognitive endpoints. Consequently, evidence was synthesized narratively using thematic analysis to identify recurring mechanistic pathways, intervention strategies, and research priorities.

Qualitative extraction focused on values relevant to clinical inferences. Hemodynamic modeling studies were coded for blood-volume assumptions, vascular stiffness, and cerebral perfusion; a 30% decrease in blood volume and a 50-100% increase in arterial stiffness were considered stressful events that may affect CBF [18]. A systematic review found that benchmarking the extent to which current therapies assess cardiovascular function, quality of life, or cognitive function is important, as treatment efficacy depends on what is measured [19]. This enabled the review to assess the plausibility of mechanisms and trial measurement.

Evidence Mapping Framework

Evidence mapping was applied to show the relationships among mechanisms, symptoms, and interventions. Impaired cerebral perfusion was mapped to attention, processing speed, and executive dysfunction; beta-blockers and midodrine were mapped as indirect because they do not measure or directly restore cerebral perfusion. Data from sustained cognitive stress testing were classified as neurovascular-stress evidence because persons with POTS had greater decreases in CBF velocity than controls under cognitive stress. Baroreflex dysfunction was mapped to executive dysfunction and reaction-speed impairment, with transcutaneous vagus nerve stimulation (tVNS) coded as a potential but not sufficiently trialed treatment. Autoimmunity and microclots were mapped to chronic brain fog, tissue hypoxia, and neural dysfunction, with plasma exchange, immunoglobulin, and fibrinolytics identified as potential treatments. Functional impairment was mapped to occupational therapy, pacing, chunking, and memory aids.

**Figure 2:**
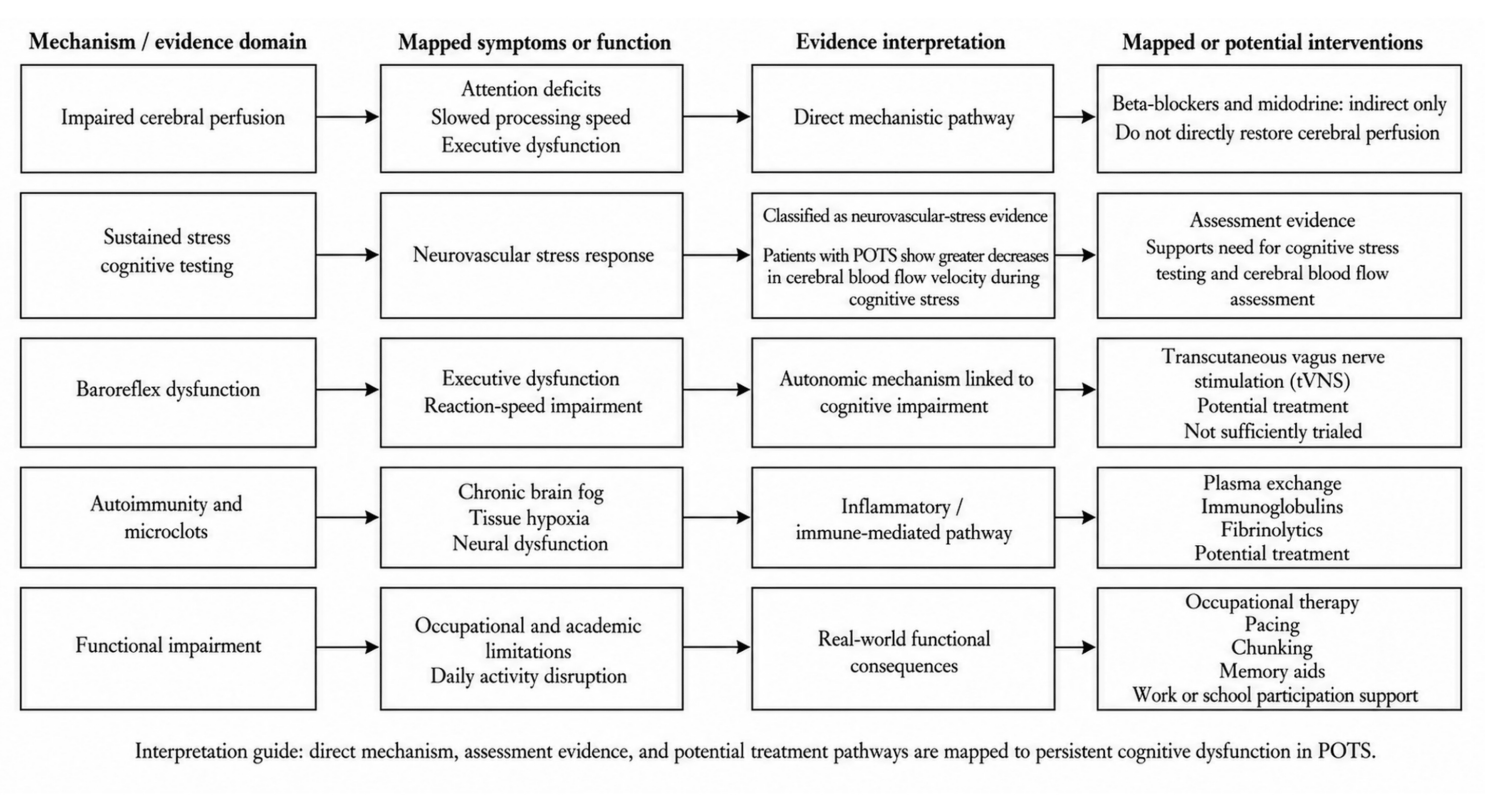
Evidence map for persistent cognitive dysfunction in POTS. Created by the author using Microsoft PowerPoint, based on a synthesis of the included studies. POTS: Postural orthostatic tachycardia syndrome.

Figure 2 presents an evidence-mapping model that connects key mechanisms of persistent cognitive dysfunction in POTS with symptom characteristics, evidence interpretations, and possible interventions. It identifies impaired cerebral perfusion as being associated with attention, processing speed, and executive deficits, and beta-blockers and midodrine as indirect treatments. It also connects cognitive stress testing to evidence of neurovascular stress; baroreflex dysfunction to tVNS; autoimmunity and microclots to evidence of plasma exchange, immunoglobulins, and fibrinolytics; and functional impairment to occupational therapy, pacing, chunking, and memory aids as practical rehabilitation for cognitive recovery and symptom control in clinical situations.

Quality Appraisal and Risk-of-Bias Assessment

Risk of bias was evaluated according to study design. Randomized controlled trials were assessed using RoB 2-informed domains; systematic reviews were assessed using ROBIS-informed criteria; and observational studies, case-control investigations, cohort studies, and case series were assessed using Joanna Briggs Institute (JBI) critical appraisal tools. Studies were evaluated for participant selection, diagnostic certainty, comparator adequacy, outcome measurement, confounding, completeness of follow-up, selective reporting, and direct applicability to persistent cognitive dysfunction in POTS.

Because this review synthesized heterogeneous evidence, including mechanistic imaging studies, physiological challenge studies, systematic reviews, clinical trials, case reports, case series, and rehabilitation studies, studies were not excluded solely because they had a high risk of bias. Instead, the risk-of-bias assessment was used to determine the strength of inference. Evidence with low or moderate risk of bias was treated as stronger support for mechanistic interpretation, whereas uncontrolled case reports, case series, and emerging microclot or immunotherapy literature were interpreted as hypothesis-generating rather than confirmatory [20].

Statistical and Practical Interpretation Strategy

The statistical interpretation approach focused on effect-quantifying values rather than broad claims. Results were interpreted using six practical values: 61% abnormal CBF on SPECT; 51% variance in health utility explained by abnormal CBF regions plus autonomic and gastric symptoms; 7.8% vs 1.8% CBF velocity reductions during cognitive stress; 48 +/- 11 vs 55 +/- 10 executive-function t-scores; 48% baseline symptom variance explained by psychosocial factors; and 4% six-month symptom variance explained by hemodynamic measures. These values could not be statistically pooled because the review included diverse studies, but they were incorporated into the assessment of whether current treatments, measures, and trial designs were well aligned with the mechanisms of POTS-related cognitive dysfunction. Thus, the ultimate interpretation focused on clinical fit: whether the evidence supports cognitive assessment, subtype classification, and the development of multimodal therapy.

Results and discussion

Although cerebral hypoperfusion and autonomic dysfunction currently have the strongest mechanistic support, the overall body of evidence remains heterogeneous. Immune dysregulation, mast cell activation, fibrinaloid microclots, and inflammatory mechanisms remain plausible contributors to persistent cognitive dysfunction but require validation in prospective controlled studies. Accordingly, these findings should be interpreted as hypothesis-generating rather than definitive causal mechanisms.

Cognitive Dysfunction in POTS Is Not Fully Explained by Tachycardia

In the studies reviewed, sustained subjective cognitive impairments in POTS cannot be fully explained by orthostatic tachycardia. Although orthostatic tachycardia is the main diagnostic feature, patients commonly complain of brain fog, slowed thinking, word-finding difficulty, and loss of concentration and productivity, even when orthostatic tachycardia is partially treated. This finding supports viewing POTS as not only a cardiovascular condition but also a neurological and cerebrovascular state. In practice, a clinic that only measures standing pulse, blood pressure, and the severity of dizziness may disregard one of the symptoms most strongly linked to education, career loss, and loss of independence. The response to treatment, therefore, should differentiate between cardiovascular and cognitive improvement, as the former may not predict improvement in the latter.

Cerebral Hypoperfusion Provides a Strong Mechanistic Explanation for Brain Fog

Cerebral perfusion results provide the strongest objective evidence in support of this interpretation. When evaluating the brains of 56 POTS patients with cognitive dysfunction using SPECT, cerebrovascular abnormalities were present in 61% of patients, including perfusion changes while patients were supine, indicating that poor brain blood flow is not exclusively orthostatic [21]. Perfusion abnormalities in the lateral prefrontal cortex are relevant to complaints of attention, working memory, inhibition, and executive control, and altered sensorimotor perfusion may affect cognitive-motor processing speed. The implication is that brain fog cannot be overlooked in patients who have abnormal orthostatic vital signs. Instead, cognitive testing and brain perfusion assessment, or referral to neurology, autonomic medicine, or neurorehabilitation, may be useful for these patients.

The sustained cognitive stress data shed light on this process, showing that cognitive stress may also increase the vulnerability of CBF. In a cognitive stress protocol, the mean decrease in CBF velocity among POTS subjects was 7.8%, versus 1.8% in controls [22]. This difference has clinical implications, as many cognitively demanding activities are performed while sitting, such as exams, computer use, shift work, and driving. Therefore, cognitive deterioration in POTS may be due to the combination of metabolic demand and compromised cerebrovascular control. Furthermore, findings indicate that future cognitive testing should assess cognitive performance under more realistic paradigms, such as cognitive load while seated, prolonged attention, and postural change.

A brain-based model of cerebral hypoperfusion as a mechanism may help explain brain fog in POTS. It indicates decreased perfusion in the lateral prefrontal and sensorimotor areas, which were associated with attention deficits, impaired working memory, executive dysfunction, slower processing speed, and poor cognitive-motor integration. The figure presents SPECT evidence from 56 patients, with 61% exhibiting abnormal CBF on supine imaging. It highlights how chronic brain fog can reflect underlying neurovascular dysfunction that requires cognitive screening, neurovascular evaluation, and functional outcome measurement in clinical practice to provide targeted care.

Autonomic Dysregulation Contributes to Acute and Chronic Cognitive Impairment

Autonomic dysregulation continues to play a prominent role in explaining the link between cardiovascular fluctuations and cognition. Excessive sympathetic activation, catecholamine variations, dysfunctional baroreflex buffering, sleep disturbance, and hypervigilance may have a long-term impact on systems in the prefrontal cortex involved in flexible attention and planning. Research on psychological and cognitive issues in POTS indicates that these issues are interrelated, and that symptoms of anxiety, depression, autonomic arousal, and cognitive impairment may aggravate each other [23]. This should not imply that cognitive dysfunction is psychogenic. Rather, it implies that psychological stress and autonomic physiology may bidirectionally affect each other. For patient care, the history related to POTS should include postural cognitive symptoms, sleep quality, anxiety screening, drug effects, and functional tests, which may influence whether cognitive dysfunction manifests as mild, variable, or severe.

Neuroinflammation and Autoimmunity May Explain Persistent Symptoms in Subgroups

Additional support for autoimmune or inflammatory subgroups of POTS comes from the fact that some patients experience persistent cognitive symptoms despite treatment. Common treatment options that may alter heart rate or blood pressure without eliminating autoantibodies, lessening brain inflammation, or unblocking small vessels include beta-blockers, ivabradine, and midodrine. Recently, a randomized trial (iSTAND) was conducted using intravenous immunoglobulin (IVIG) to treat autoimmune POTS (n = 30), with no significant benefit over albumin, although both groups showed improvement that may be attributed to volume loading from intravenous therapy [24]. Such a finding should not halt inquiry into immunotherapy. Instead, it emphasizes that future research should improve subtype selection, include cognition-specific outcomes, use biomarkers, and include control arms that separate the effects of immune modulation from those of volume expansion. While this null result constrains enthusiasm for IVIG as a straightforward treatment, it is informative rather than definitively negative: the absence of a cognitive primary endpoint, the small sample size, and the volume expansion confound collectively identify what a more rigorous future trial would need to address, rather than establishing immunotherapy as ineffective.

The most directly relevant evidence to the cognitive dimension of this research question in the source set reviewed here is provided by the first published case of plasma exchange producing documented improvement in cognitive function in a patient with long-COVID-related POTS and autoimmune neurological dysfunction (Seeley MC et al., 2023), establishing proof of concept for the cognitive-immunotherapy link even within the evidentiary constraints of a case report. Further supporting autoimmune subtype identification as the critical patient selection variable, symptom improvement was documented after subcutaneous immunoglobulin and plasmapheresis were administered in POTS patients with suspected autoimmune etiologies (Kesterson K et al., 2022). Plasmapheresis, plasma exchange, and fibrinolytic therapies are being considered for post-infectious or autoimmune variants, particularly when neurological signs, including cognitive impairment, are present.

Emerging Interventions Require Cognitive Endpoints and Multimodal Design

The overall evidence suggests that this heterogeneous syndrome is unlikely to be resolved by single therapies aimed at protracted brain fog. The most appropriate solution would involve combining mechanism-based diagnostics with a multimodal model: orthostatic intolerance would be addressed through orthostatic stability, sympathetic surges through autonomic stability, immune-mediated issues through immunologically targeted approaches, and cognitive retraining through improved cognitive functioning in real-life situations. A narrative review of observational and interventional studies showed that POTS symptoms are multifaceted and heterogeneously assessed, underscoring why studies that focus only on general symptoms or orthostasis cannot determine whether cognition changes [25]. Thus, cognition should be a primary or secondary outcome in POTS trials.

Table 2 summarizes new intervention pathways for persistent cognitive dysfunction in POTS by cross-tabulating each identified option with its target mechanism, recommended endpoints, and clinical practice implications. It indicates that tVNS targets autonomic imbalance, whereas immune-targeted approaches are used for autoimmune mechanisms. Rehabilitation based on occupational therapy is also focused on functional recovery, achieved through pacing, task segmentation, memory aids, and graded return-to-work or school planning. The table also highlights multimodal monitoring, including cardiovascular and cognitive measures reviewed every 6 to 12 weeks in specialty POTS clinical practice.

**Table 2:** Emerging multimodal POTS interventions, target mechanisms, recommended endpoints, and clinical monitoring applications. tVNS: Transcutaneous vagus nerve stimulation; POTS: Postural orthostatic tachycardia syndrome.

Emerging intervention or clinical component	Targeted mechanism or purpose	Recommended cognitive/autonomic endpoints	Practical clinical application	Source(s)
Transcutaneous vagus nerve stimulation	Targets autonomic imbalance by increasing parasympathetic tone and reducing sympathetic dominance	Resting heart rate, standing heart rate, blood pressure, attention, executive function, fatigue severity, and quality of life	Could be tested as an autonomic-modulation intervention in patients whose cognitive dysfunction appears linked to sympathetic overactivation	[17]
Immunotherapy, plasma exchange, or fibrinolytic approaches	Targets autoimmune, inflammatory, post-viral, or microvascular contributors to persistent brain fog	Cognitive fatigue, patient-reported brain fog severity, working memory, processing speed, inflammatory or autoimmune markers, and functional recovery	Most relevant for autoimmune, inflammatory, or long-COVID-related POTS subtypes after mechanism-based screening; these approaches remain emerging and require careful patient selection	[8,15,20,24]
Occupational therapy-based cognitive rehabilitation	Addresses daily functional impairment through memory aids, task segmentation, pacing, environmental simplification, and graded return-to-work or return-to-school planning	School or work participation, sustained attention, executive function, task completion, cognitive fatigue, and quality of life	Can be adapted for POTS patients who report functional limitations related to brain fog, memory difficulty, slowed processing, and executive dysfunction	[12]
Multimodal trial and clinic monitoring model	Combines biological treatment, autonomic modulation, and rehabilitation instead of relying on a single-drug model	Baseline and follow-up cognitive testing, medication exposure, comorbid migraine or pain, fatigue severity, cardiovascular response, and cognitive response	A 6- to 12-week review interval can help distinguish pulse control from true cognitive improvement and guide staged treatment decisions	[12,17,20,24,25]

Three areas of intervention are likely most important. tVNS may improve vagal tone and reduce sympathetic predominance, but it still requires further testing in randomized trials with cognitive outcomes. Immunotherapy, plasma exchange, or fibrinolytic treatment may also be most relevant for autoimmune or long-COVID-related subtypes, but the available studies are small-sampled and have variable protocols. Additionally, occupational therapy-based cognitive rehabilitation is immediately applicable to provide memory compensation, pacing, task segmentation, accommodations, school supports, and return-to-work planning [26]. The key take-home message is that, rather than focusing on response definitions based on heart rate, future studies should consider composite outcomes that include measures of cerebral perfusion, sympathetic hyperactivity, cognition, self-reported brain fog, and school or work participation. This could be applied in a specialty autonomic clinic, since a single visit can include orthostatic blood pressure, drug exposures, fatigue severity, short attention tests, whether automated or tablet-based, and structured participation goals. This would allow differentiation between hemodynamic recovery and cognitive gains on follow-up, respectively, along with self-reported participation measures.

Future trial planning and risk-of-bias interpretation should also consider adjacent methodological evidence relevant to POTS cognition research. Wearable POTS monitoring may help contextualize symptoms outside clinic visits [27], multimodal cognitive trial designs may guide complex intervention planning [28], occupational therapy cognitive-intervention literature may inform functional rehabilitation models [29], microclot biomarker work may support future inflammatory or long-COVID-related POTS stratification [30], and mast-cell/basophil activation testing literature may help guide immune-phenotyping strategies when mast-cell activation is clinically suspected [31].

Risk-of-Bias Assessment of Included Evidence

A design-specific risk-of-bias assessment was performed according to study type, including appraisal of sample selection, diagnostic certainty, control-group status, objective cognitive testing, objective physiological measurement, follow-up completeness, confounding, and directness to POTS-related cognitive dysfunction. The full risk-of-bias assessment is summarized in Table 3.

**Table 3:** Risk-of-bias assessment of included evidence. POTS: Postural orthostatic tachycardia syndrome; RoB 2: Cochrane Risk of Bias 2 tool; ROBIS: Risk of Bias in Systematic Reviews; JBI: Joanna Briggs Institute; SPECT: Single-photon emission computed tomography; IVIG: Intravenous immunoglobulin; SCIG: Subcutaneous immunoglobulin; tVNS: Transcutaneous vagus nerve stimulation.

Source	Study type / evidence domain	Appraisal approach	Key risk-of-bias concerns	Overall risk of bias	Interpretation in this review
Seeley MC et al. [21]	Observational SPECT imaging study in POTS with cognitive dysfunction	JBI-informed observational appraisal	Referral/sample-selection bias; no longitudinal causal confirmation; imaging findings may not generalize to all POTS phenotypes	Moderate	Strong direct evidence linking POTS-related cognitive dysfunction with abnormal cerebral perfusion
Wells R et al. [22]	Controlled physiological cognitive-stress study	JBI-informed case-control/physiological appraisal	Small sample; laboratory task may not fully represent daily cognitive load; limited long-term follow-up	Moderate	Direct evidence that cognitive stress can worsen cerebral blood flow velocity and cognitive performance in POTS
Miller AJ et al. [6]	Controlled active-standing cognitive study	JBI-informed case-control appraisal	Possible performance bias; limited blinding; posture-specific testing may not capture all brain-fog states	Moderate	Supports impairment of attention and executive function during orthostatic challenge
Shanks L et al. [16]	Review/theoretical synthesis of cognitive impairment in CFS and POTS	ROBIS-informed relevance appraisal	Indirectness because evidence includes CFS as well as POTS; limited POTS-specific intervention data	Moderate to high	Useful background evidence, but not sufficient alone for treatment conclusions
Raj V et al. [23]	Narrative review of cognitive and psychological issues in POTS	ROBIS-informed narrative-review appraisal	Non-systematic review design; possible selective inclusion of evidence; heterogeneous cognitive measures	Moderate	Useful contextual evidence for brain fog and psychological-autonomic interaction
Wei L et al. [18]	Hemodynamic modeling study	Mechanistic appraisal	Model assumptions may not reflect all clinical phenotypes; limited direct cognitive testing	Moderate to high	Supports biological plausibility of hypovolemia and vascular dysfunction affecting cerebral perfusion
Mueller BR et al. [7]	Observational study of cardiovagal baroreflex sensitivity and headache/POTS	JBI-informed observational appraisal	Pain/headache population may introduce confounding; wide confidence intervals; cognition not the primary outcome	Moderate to high	Supports an autonomic-baroreflex contribution but remains indirect for cognition
Pierson BC et al. [9]	Systematic review of oral medications for POTS	ROBIS-informed systematic-review appraisal	Heterogeneous medication studies; many outcomes cardiovascular rather than cognitive; limited randomized data	Moderate	Indicates that pharmacological evidence focuses mainly on heart rate and symptoms, not cognition
Wells R et al. [19]	Systematic review/meta-analysis of POTS therapies	ROBIS-informed systematic-review appraisal	Heterogeneity of interventions and outcomes; cognitive endpoints rarely reported	Moderate	Supports the conclusion that existing treatment evidence is not cognition-centered
Knoop I et al. [11]	Longitudinal symptom-severity study	JBI-informed cohort appraisal	Self-reported symptoms; participants were under investigation for POTS; possible attrition and confounding	Moderate	Supports persistence of symptom burden and the limited explanatory value of hemodynamic measures alone
Knoop I et al. [25]	Narrative review of symptom burden	ROBIS-informed narrative-review appraisal	Heterogeneous symptom definitions; variable study quality; cognition inconsistently measured	Moderate to high	Supports the need for standardized symptom and cognitive outcomes
Hollingsworth C et al. [12]	Participant-identified cognitive challenges and strategies	JBI-informed qualitative/observational appraisal	Self-report design; no randomized intervention; limited generalizability	Moderate to high	Clinically useful for identifying functional cognitive limitations and rehabilitation targets
Oetjen L et al. [32]	Retrospective interdisciplinary rehabilitation outcomes	JBI-informed retrospective appraisal	No randomized control; chart-review design; perceived progress may be subject to reporting bias	High	Supports the feasibility of functional rehabilitation goals but not definitive efficacy
Tran HH et al. [17]	Review of noninvasive vagus nerve stimulation	ROBIS-informed review appraisal	Evidence is largely indirect for POTS cognition; randomized cognitive endpoints are lacking	High	Identifies tVNS as a potential intervention requiring controlled trials
Vernino S et al. [24]	Randomized controlled trial of IVIG for presumed autoimmune POTS	RoB 2-informed appraisal	Small sample; albumin comparator may create volume-expansion confounding; cognition not the primary endpoint	Some concerns / moderate	Provides the highest-level immune-therapy evidence but does not prove cognitive benefit
Kesterson K et al. [8]	Case series of SCIG or plasmapheresis	JBI case-series appraisal	Uncontrolled design; small sample; retrospective symptom assessment; selection bias	High	Hypothesis-generating evidence for autoimmune POTS subgroups
Seeley MC et al. [15]	Case report of plasma exchange and cognitive improvement	JBI case-report appraisal	Single-patient evidence; no control; possible placebo effect, regression, or concurrent-treatment effects	High	Proof of concept only; cannot support a broad treatment recommendation
Kell DB et al. [20]	Mechanistic review of fibrinaloid microclots in POTS/long COVID	ROBIS-informed mechanistic-review appraisal	Emerging pathway; indirect treatment evidence; biomarker standardization lacking	High	Hypothesis-generating support for microvascular/inflammatory mechanisms

The risk-of-bias assessment indicates that the strongest direct evidence for persistent cognitive dysfunction in POTS comes from controlled physiological and imaging studies assessing cerebral perfusion, CBF velocity, attention, and executive function. In contrast, immune-directed therapy, plasma exchange, fibrinolytic mechanisms, tVNS, and occupational-therapy-based cognitive rehabilitation remain promising but methodologically limited because much of the available evidence is uncontrolled, indirect, or not designed with cognition as the primary endpoint. Therefore, this review interprets these approaches as research priorities rather than established treatments.

Future research recommendations

Develop and Validate a POTS-Specific Cognitive Outcome Battery

Future research should develop a cognitive outcome battery for POTS-related brain fog. This should measure sustained attention, working memory, processing speed, executive function, word retrieval, cognitive exhaustion, and workplace performance. It should include patient-reported impairment, objective neuropsychological tasks, and a posture-based challenge in supine, seated, and upright positions. Wearable monitoring should also be considered, as it can provide 24-hour heart rate, activity, sleep, and symptom data to contextualize cognitive fluctuations between laboratory visits [27]. A pragmatic test design would involve 200 to 300 diagnosed POTS patients, representing diagnostic subtypes, and 100 age- and sex-matched controls. The long-term POTS survey reveals variability in diagnosis, medication exposure, and clinical outcomes, so questionnaire batteries should also include treatment history, illness duration, comorbidities, and school or work attendance.

Design Multimodal Cognitively Instrumented Trials

Trials should adopt shared cognitive outcomes and move away from single-endpoint cardiovascular designs. A feasible POTS trial could compare standard cardiovascular care with a multimodal approach that includes autonomic stabilization, subtype-guided biological treatment when appropriate, and occupational-therapy-based cognitive rehabilitation. Primary outcomes should include objective attention, working memory, processing speed, executive function, patient-reported brain fog severity, cognitive fatigue, and school or work participation. Secondary outcomes should include standing heart rate, blood pressure, orthostatic symptom burden, fatigue, quality of life, medication exposure, inflammatory or autoimmune markers when relevant, and adverse events.

The iSTAND randomized controlled trial found that IVIG did not clearly outperform albumin in presumed autoimmune POTS, which highlights the need to distinguish immune effects from volume-expansion effects and to include cognition-specific endpoints in future immune-therapy trials [24]. Case-series and case-report evidence involving subcutaneous immunoglobulin, plasmapheresis, or plasma exchange should therefore be treated as hypothesis-generating rather than definitive [8,15]. Similarly, tVNS should be studied as a potential autonomic-modulation intervention with cognitive endpoints rather than assumed to improve brain fog without direct testing [17]. Occupational-therapy-based strategies, including pacing, memory aids, task segmentation, environmental simplification, and graded return-to-school or return-to-work planning, should be integrated as functional interventions because patients identify these strategies as directly relevant to daily cognitive impairment [12,32].

Stratify Patients by POTS Subtype and Biomarker Profile

Researchers should stratify POTS patients before treatment rather than assuming that all patients have the same POTS phenotype. Although other subtypes may exist, hypovolemic, neuropathic, hyperadrenergic, autoimmune, and long-COVID-associated POTS are recommended subtypes for classification. This is necessary because pathogenesis can differ between patients, and a given treatment may be helpful, ineffective, or even harmful in one patient but not in another. At the molecular level, fibrinaloid microclot complexes may help distinguish pre-COVID POTS, Long COVID, and Long COVID-POTS and, therefore, may support biomarker-driven classification [30].

Measures of CBF, baroreflex activity, adrenergic receptor autoantibodies, indices of inflammation, microclot load, and mast cell activity are examples of possible biomarkers. In situations involving possible allergy or suspected mast cell or basophil activation, the physiology of mast cell or basophil activation can guide the selection of laboratory tests to detect biomarkers of immune activation [31]. Stratification will reduce noise, improve identification of affected responders, and facilitate more accurate treatment plans.

Integrate Occupational Therapy and Real-World Functional Outcomes

Future research should develop a POTS-specific occupational therapy protocol for cognitive dysfunction. The protocol should include pacing, scheduled cognitive breaks, task segmentation, compensatory memory strategies, environmental modification, graded return-to-school or return-to-work planning, posture-aware task scheduling, and strategies to manage cognitive fatigue during reading, screen use, driving, studying, and work. These interventions should be evaluated using both objective and functional outcomes, including sustained attention, processing speed, executive function, cognitive fatigue, school or work attendance, task completion, quality of life, and patient-defined participation goals.

Real-world participation outcomes are essential because POTS-related cognitive dysfunction affects education, employment, independence, and daily routines. Participant-centered POTS research has already identified cognitive barriers and compensatory strategies that can guide intervention development [12]. Interdisciplinary rehabilitation data also show that functional goals can be documented in adolescents and young adults with POTS [32]. Objective cognitive-fatigue measurement should also be included because real-time objective measures can supplement self-report when return-to-school, return-to-work, or return-to-activity outcomes are studied [33]. For students and workers with POTS, future studies should also track digital workload, screen exposure, task switching, and cognitive overload because these factors may worsen participation in cognitively demanding school or workplace settings [34].

Future investigations should employ validated POTS-specific cognitive outcome batteries incorporating objective assessments of attention, executive function, processing speed, working memory, cognitive fatigue, and real-world occupational or educational functioning. Randomized clinical trials should integrate cognitive outcomes alongside cardiovascular, autonomic, inflammatory, and patient-reported measures to determine whether therapeutic interventions improve cognition independently of orthostatic symptom control.

## Conclusions

Persistent cognitive dysfunction represents an important yet incompletely understood manifestation of POTS. Current evidence suggests that cerebral hypoperfusion, autonomic dysregulation, and potentially immune-mediated mechanisms contribute to brain fog and executive dysfunction; however, the available evidence remains predominantly observational and mechanistic. Consequently, causal inferences cannot yet be established. Prospective studies using standardized cognitive outcome measures and multimodal intervention designs are required to clarify mechanisms and improve patient care.

These findings support a broader understanding of POTS that extends beyond cardiovascular dysfunction. Current therapeutic strategies primarily target orthostatic intolerance and tachycardia, whereas cognitive outcomes remain underrecognized and are infrequently evaluated in clinical studies. The absence of standardized cognitive assessment tools for POTS further limits the ability to determine treatment effects on neurocognitive function. Incorporating validated cognitive measures into future research may improve the evaluation of interventions and better reflect patient-reported functional impairment.

Future studies should investigate the relationships among CBF, autonomic function, inflammatory and immune biomarkers, and objective cognitive performance across different POTS subtypes. Multidisciplinary approaches integrating autonomic management with targeted cognitive rehabilitation may offer additional benefits, although high-quality prospective studies are required to establish their effectiveness. A more comprehensive framework that includes both cardiovascular and neurocognitive outcomes may improve the assessment and management of patients with POTS.
